# Development of a mesoscopic framework spanning nanoscale protofibril dynamics to macro-scale fibrin clot formation

**DOI:** 10.1098/rsif.2021.0554

**Published:** 2021-11-10

**Authors:** Naoki Takeishi, Taiki Shigematsu, Ryogo Enosaki, Shunichi Ishida, Satoshi Ii, Shigeo Wada

**Affiliations:** ^1^ Graduate School of Engineering Science, Osaka University, 1-3 Machikaneyama Toyonaka, Osaka 560-8531, Japan; ^2^ Graduate School of Engineering, Kobe University, 1-1 Rokkodai, Nada, Kobe, 657-8501, Japan; ^3^ Graduate School of Systems Design, Tokyo Metropolitan University, 1-1 Minami-Osawa Hachioji, Tokyo 192-0397, Japan

**Keywords:** fibrin clot, protofibril, mesoscopic dynamics, computational modelling

## Abstract

Thrombi form a micro-scale fibrin network consisting of an interlinked structure of nanoscale protofibrils, resulting in haemostasis. It is theorized that the mechanical effect of the fibrin clot is caused by the polymeric protofibrils between crosslinks, or to their dynamics on a nanoscale order. Despite a number of studies, however, it is still unknown, how the nanoscale protofibril dynamics affect the formation of the macro-scale fibrin clot and thus its mechanical properties. A mesoscopic framework would be useful to tackle this multi-scale problem, but it has not yet been established. We thus propose a minimal mesoscopic model for protofibrils based on Brownian dynamics, and performed numerical simulations of protofibril aggregation. We also performed stretch tests of polymeric protofibrils to quantify the elasticity of fibrin clots. Our model results successfully captured the conformational properties of aggregated protofibrils, e.g., strain-hardening response. Furthermore, the results suggest that the bending stiffness of individual protofibrils increases to resist extension.

## 1. Introduction

Fibrin clots are one of dominant components of thrombi (others are, e.g. red cells or platelets), which play a crucial role in events such as haemostasis [1] and pulmonary thromboembolism [2]. Studies of fibrin clots have been conducted to clarify both physiological and pathological significance [1,3]. Particular attention has been paid to multi-scale spatiotemporal processes connecting the small components of fibrin clots (e.g. fibrinogen) to macro-scale thrombi [4–7]. Fibrin clots themselves consist of components on multiple scales. Each clot, over several microns in size, is the product of protofibril aggregation mediated by fibrinogen, which is a nano-scale 45-nm-long plasma protein consisting of six paired polypeptide chains (two pairs each of A*α*-chains, B*β*-chains and *γ*-chains) [8–10]. Fibrinogen is activated by thrombin and polymerizes into a double-stranded fibre, the so-called protofibril [1,10]. Architecturally, the protofibril is a regularly repeating 22.5-nm unit corresponding to one-half the length of the fibrinogen protein [10–12].

Since a fibrin clot is an interlinked, branching structure consisting of protofibrils [13], it is expected that clot elasticity can result from the reaction of the polymeric filaments between cross-links, from alterations in the network structure, or both [7,14]. Researchers have shown that the mechanical properties of the fibrin network structure are altered by chemical and mechanical conditions (e.g. fibrinogen concentrations and the solvent flow field) [6,13,15,16]. For instance, it is well known that factor XIIIa increases the elasticity of individual fibrin fibres [17], thereby enhancing the stability of clots by increasing their stiffness and resistance against deformation [13,18,19]. It is also known that some medications commonly used to prevent and treat cardiovascular diseases (e.g. aspirin and heparin) affect fibrin polymerization and clot structure, making fibrin more porous, permeable, and susceptible to lysis [20,21]. Piechocka *et al.* [22] recently showed that *γ*-chain cross-linking contributes to clot elasticity by changing the force-extension behaviour of protofibrils, whereas *α*-chain cross-linking stiffens the clot, as a consequence of tighter coupling between the constituent protofibrils [22]. Despite a number of studies of fibrin clot formation, much is still unknown, in particular about how nanoscale protofibril dynamics affect macro-scale fibrin clot formation and then clot mechanical properties. Considering that the clotting time (or gel point) has been used in clinical assays as an indication of altered coagulation, understanding the mechanisms of fibrin polymerization may provide a basis for informative diagnostic tools, such as molecular markers of thrombin generation and intravascular fibrin deposition.

To comprehensively investigate the fibrin clot architecture and its mechanical properties, Onck *et al.* [23] numerically investigated the dynamics of individual (actin) fibrous components under shear flow using a two-dimensional model, and showed that stiffening of non-affine, cross-linked semiflexible networks is caused by the transition of a bending-dominated response at small strains to a stretching-dominated response at large strain [23]. Moiseyev *et al.* [24] used a theoretical and statistical model of fibrin clot growth to quantify the relationship between the density of fibre cross-linking and clot elasticity [24]. These models, however, cannot fully deal with the dynamics of fibrin clot components. Yesudasan *et al.* proposed the coarse-grained molecular dynamics model to investigate the complex network of fibrin clots [25,26]. However, the model focuses on the dynamics of fibrinogen molecules on a scale of several hundreds nanometres, whereas the network structures of fibrin clots on the scale of several micrometres, corresponding to the cellular scale level, have not yet been fully described.

A mesoscopic framework is needed to clarify the spatio-temporal relationship between the nanoscale protofibril behaviours and the mechanical properties of micrometre-scale fibrin clots, but no such framework has been established yet. Therefore, the objective in this study was to develop a framework to investigate fibrin clot formation on multiple scales, with a focus on nanoscale protofibrils behaviour. We proposed a minimal mesoscopic model for protofibrils based on Brownian dynamics. Using this model, we performed simulations of protofibril aggregation. For these polymeric protofibrils, we also performed numerical stretch tests to quantify the elasticity of fibrin clots. Based on these numerical approaches, we discuss the feasibility of a proposed model to study the multi-scale relationship between protofibril molecular dynamics and the mechanical response of fibrin clots.

## 2. Methods

### 2.1. Coarse-grain protofibril model

**Figure 1.**
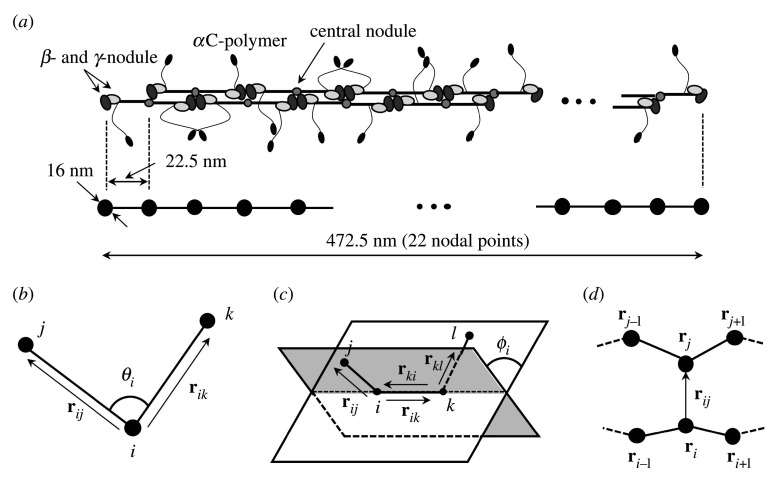
(*a*) Schematic of a protofibril model with length 472.5 nm, consisting of 22 nodal points in each 22.5-nm-long segment (node to node). Schematic images of (*b*) bending, (*c*) torsion and (*d*) aggregation energy, where superscript (·) represents two adjacent protofibrils.

We consider the dynamics of protofibrils with a length of 472.5 nm in a static medium. Based on electron microscope observations [19], each protofibril is modelled as 22 nodal points in series, where the length of the segment consisting of two nodal points is set to 22.5 nm [11], which is the half the length of the 45-nm-long fibrinogen molecule [27]. The model schematic is shown in figure 1(*a*). Each nodal point is governed by the following overdamped Langevin equation:

2.1 $$c\frac{\partial r_{i}}{\partial t}=-\frac{\partial W}{\partial r_{i}}+F_{i}^{\mathrm{rand}},$$

where **r**_*i*_ is the position of the *i*th node, *W* is the total potential energy in the computational domain, $F_{i}^{\mathrm{rand}}$ is the random force of the *i*th node, characterized as thermal fluctuations of solvent molecules and *c* is the frictional coefficient estimated by Stokes’ Law, i.e. *c* = 6*πμa*. Here, *μ* (= 1.2 mPa · s) is the solvent viscosity of human plasma. Based on transmission electron microscopy, atomic force microscopy (AFM) and X-ray crystallographic data, the fibrinogen molecule is approximately 2–7 nm in diameter [28–31]. Recent experimental observations using AFM reported that the length of *γ*-nodule is 5 nm in the naturally folded state (i.e. force-free condition). Considering a double-stranded fibre of protofibril [1,10], we define the characteristic radius of fibrinogen as 8 nm.

In this study, the total potential energy *W* is expressed as five different components, specifically stretch *W*^*S*^, bend *W*^*B*^, torsion *W*^*T*^, aggregation *W*^*A*^ and repulsive energy *W*^*R*^

2.2 $$W=W^{S}+W^{B}+W^{T}+W^{A}+W^{R}.$$

The stretch energy *W*^*S*^ and stretch force $f_{i}^{S}$ of the *i*th node can be expressed as

2.3*a* $$W^{S}=\sum_{ij}\frac{k^{S}}{2}{\left(|r_{ij}|-r_{0}^{S}\right)}^{2},$$

2.3*b* $$f_{i}^{S}=-\frac{\partial W^{S}}{\partial r_{i}}=k^{S}(|r_{ij}|-r_{0}^{S})\frac{r_{ij}}{\left|r_{ij}\right|}$$

2.3*c* $$\;\mathrm{and}\;r_{ij}=r_{j}-r_{i},$$

where *k*^*S*^ is the stretch energy constant, $r_{0}^{S}$ (= 22.5 nm) is the reference length, which is half the length of the fibrinogen molecule [11], and **r**_*j*_ is the *j*th node adjacent to the *i*th node in the same protofibril. From both AFM experiments and molecular dynamics simulations, the stretch energy constant can be estimated from the extension dynamics of a single fibrinogen molecule [10,32]. For instance, Lim *et al.* [32] estimated the longitudinal spring constant of protofibrils as *O*(*k*^*S*^) ∼ 10^−3^ N m^−1^ [32]. While in more recent coarse-grained molecular simulations by Tan *et al.* [33], the force constant for neighbouring bonds corresponding to *k*^*S*^ in this study, was set to be 250 kJ mol^−1^ Å^−2^, which can be rewritten as 0.42 N m^−1^ [33]. Hence, in this study, we set as *k*^*S*^ = 0.01 N m^−1^.

The bending energy *W*^*B*^ and bending force $f_{i}^{B}$ of the *i*th node between the *j*th and *k*th nodes can be expressed as

2.4*a* $$W^{B}=\sum_{ijk}\frac{k^{B}}{2}{\left(\theta_{ijk}-\theta_{0}\right)}^{2},$$

2.4*b* $$\theta_{ijk}=\text{arccos}\left(\frac{r_{ij}\cdot r_{ik}}{\left|r_{ij}||r_{ik}\right|}\right)$$

2.4*c* $$\;\mathrm{and}\;f_{i}^{B}=-\frac{\partial W^{B}}{\partial r_{i}},$$

where *k*^*B*^ is the bending energy constant, *θ*_*ijk*_ is the angle at the *i*th node between the *j*th and *k*th nodes (figure 1*b*), and *θ*_0_ (=*π*) is the reference angle [10]. In previous coarse-grained molecular dynamics simulations by [25,33], the bending energy constant was used in the range between 10^−19^ and 10^−17^ J rad^−2^. Hence, in this study, the bending energy constant is set as *k*^*B*^ = 0.1–10 × 10^−18^ J rad^−2^. The final form of equation (2.4*c*) is described in appendix A.

The torsion energy *W*^*T*^ and torsion force $f_{i}^{T}$ of the *i*th node can be expressed as

2.5*a* $$W^{T}=\sum_{ijkl}\frac{k^{T}}{2}{\left(\phi_{i}-\phi_{0}\right)}^{2}$$

2.5*b* $$\phi_{i}=\mathrm{sgn}\{r_{\;jk}\cdot (m\times n)\}\text{arccos}\left(\frac{m\cdot n}{\left|m||n\right|}\right)$$

2.5*c* $$\;\mathrm{and}\;f_{i}^{T}=-\frac{\partial W^{T}}{\partial r_{i}},$$

where *k*^*T*^ is the torsion energy constant, *ϕ*_*i*_ is the torsion angle (or dihedral angle) of the *i*th node among the *j*th, *k*th and *l*th nodes (figure 1*c*), *ϕ*_0_ (=0) is the reference torsion angle, and **m** and **n** are the normal vectors, which are defined by four adjacent nodes on two planes (figure 1*c*)

2.6*a* $$m=r_{ij}\times r_{ik}$$

and

2.6*b* $$n=r_{ki}\times r_{kl}.$$

The final forms of equation (2.5*c*) at each of the four nodes are described in the appendix A. In previous coarse-grained modelling for DNA [34] and biomoleculars [33], the energy constant in equation (2.5) was considered in the range between 10^−23^–10^−21^ J rad^−2^. Although tightly coupled studies between experiment and simulation are needed to identify the value of the constant, the torsion energy constant *k*^*T*^ was set as *k*^*T*^ = 1.0 × 10^−23^ J rad^−2^ in this study.

Two protofibrils are bound to each other at the *α*C regions [1], and hence we define the aggregation force applied to two nodal points of different protofibrils, within a threshold *a*_*th*_ ($=2r_{0}^{S}$), corresponding to the length of the fibrinogen molecule [11]. Considering two adjacent nodal points of different protofibrils (see figure 1*d*), the aggregation energy *W*^*A*^ and aggregation force $f_{i}^{A}$ of the *i*th node can be written as

2.7*a* $$W^{A}=\sum_{ij}\frac{k^{A}}{2}{\left(|r_{ij}|-r_{0}^{A}\right)}^{2},$$

2.7*b* $$f_{i}^{A}=-\frac{\partial W^{A}}{\partial r_{i}}=-k^{A}(|r_{ij}|-r_{0}^{A})\frac{r_{ij}}{\left|r_{ij}\right|}$$

2.7*c* $$\;\mathrm{and}\;f_{j}^{A}=-f_{i}^{A},$$

where *k*^*A*^ is the aggregation energy constant and $r_{0}^{A}$ (=2*a*) is the reference length between two adjacent nodes that are not present on the same protofibril. For simplicity, *W*^*A*^ is modelled as a simple harmonic potential form as shown in equation (2.7*a*). The formation could be written in light of the fact that lateral aggregation of protofibrils is responsible for local (several nanometre-scale) conformational changes [35], but this is beyond the scope of the present work. Instead of aggregation force constant between two fibrinogen, molecular interaction between platelet glycoprotein receptor GPIIb/IIIa (integrin *α*_IIb_*β*_3_) and fibrinogen have been well investigated. For instance, Litvinov *et al.* (2011) experimentally investigated thermodynamics and kinetics of bonds between GPIIb/IIIa and fibrinogen, where the molecular spring (or aggregation) constant was used as 12 pN nm^−1^ (=0.12 N m^−1^) to estimate the energy needed to dissociate *α*_IIb_*β*_3_ from fibrinogen in the long-duration state [36]. While in simulation work by [37], platelet adhesion and aggregation were modelled focusing on the interaction between the GPIIb/IIIa and its ligand, where the aggregation force constant for binding between GPIIb/IIIa and fibrinogen was set to as *k*^*A*^ = 1.0 × 10^−4^ N m^−1^. According to those studies, the aggregation energy constant was set as *k*^*A*^ = 0.2–20 × 10^−3^ N m^−1^.

Short distance between two charged particles leads to ‘bouncing off’, resulting in repulsive forces between the two, which corresponds to charged particles interacting through colloidal forces at constant surface charge. Several potential models have been proposed to represent such near-field dynamics, and the Lennard–Jones potential is one of the most classical and well-known models. This was also applied to previous coarse-grained molecular dynamics simulations of protofibrils, e.g. by [25,26]. In this study, however, we focus on developing a minimal mesoscopic model for protofibrils, and hence a non-hydrodynamic inter-node repulsive force is modelled as a simple linear function of two-nodes distance, which is defined only when the two node points on different protofibrils are within the diameter of the fibrinogen (=2*a*). This force practically allows us to avoid the prohibitively small time step needed to overcome the problem of overlapping nodes on different protofibrils. The repulsive energy *W*^*R*^ and repulsive force $f_{i}^{R}$ of the *i*th node can be expressed as

2.8*a* $$W^{R}=\sum_{ij}\frac{k^{R}}{2}{\left(|r_{ij}|-r_{0}^{R}\right)}^{2},$$

2.8*b* $$f_{i}^{R}=-\frac{\partial W^{R}}{\partial r_{i}}=-k^{R}(|r_{ij}|-r_{0}^{R})\frac{r_{ij}}{\left|r_{ij}\right|}$$

**Figure 3.**
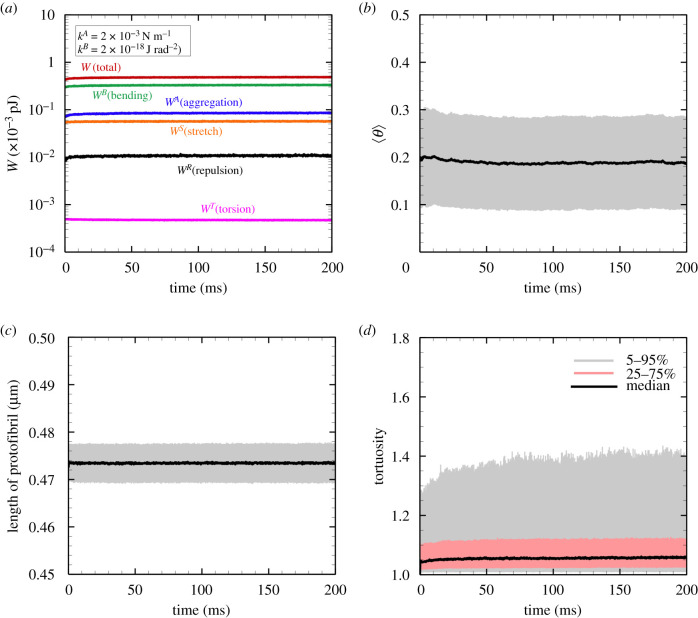
(*a*) One of the examples of the time history of each energy type; total energy *W* = *W*^*B*^ + *W*^*A*^ + *W*^*S*^ + *W*^*R*^ + *W*^*T*^ (red), bending energy *W*^*B*^ (green), aggregation energy *W*^*A*^ (blue), stretch energy *W*^*S*^ (orange), repulsive energy *W*^*R*^ (black) and torsion energy *W*^*T*^ (pink). The time histories of the mean (*b*) protofibril orientation angle and (*c*) protofibril length, where the errors represent the standard deviation of individual protofibrils (=1215). (*d*) The time history of the median of the tortuosity, where the errors represent 5–95% percentile (light grey) and 25–75% percentile (red), respectively. The results were obtained with *k*^*B*^ = 1 × 10^−18^ J rad^−2^ and *k*^*A*^ = 2 × 10^−3^ N m^−1^.

2.8*c* $$\;\mathrm{and}\;f_{j}^{R}=-f_{i}^{R}.$$

where *k*^*R*^ (=2 × 10^−4^ N m^−1^) is the repulsive resistance, and $r_{0}^{R}$ (=2*a*) is the reference length. The effect of the repulsive force on the trajectories of fibres is very small, because it changes the distance between discrete nodes only when the nodes approach within the distance corresponding to the diameter of fibrinogen, which two order of magnitude smaller than system length. Indeed, the order of the magnitude of repulsive energy was two or three orders magnitude smaller than in the total energy (figures 3*a* and 6*a*). The linear repulsive model (equation 2.8) has been also applied to cellular interaction problem, and successfully represented a cellular flow in microchannels [38].

The protofibrils experience thermal fluctuations derived both from themselves and from solvent molecules. The random force at the *i*th node is described as

2.9*a* $$F_{i}^{\mathrm{rand}}=\sigma j$$

and

2.9*b* $$\sigma^{2}=2ck_{b}T,$$

where *σ* is the magnitude of the random force, **j** is the random vector, *k*_*b*_ is the Boltzmann constant (=1.38 × 10^−23^ J K^−1^) and *T* (=300 K) is the absolute temperature in the whole system. The temporal direction satisfies the following dynamic statistics:

2.10*a* ⟨j⟩=0

and

2.10*b* $$\langle jj\rangle =I\delta (t-t^{\prime }),$$

where 〈 · 〉 represents random average procedures.

Ideally, model parameters characterizing each of the energy components in (2.2) should be determined from molecular dynamic simulations. However, this type of bottom-up approach based on energy trajectories (e.g. [39,40]) is still challenging due to the heavy computational load. In this study, we therefore proposed mesoscopic model properties to qualitatively represent the experimental observations [7,13,41], and in particular focused on the effect of the bending energy constant *k*^*B*^ and the aggregation energy constant *k*^*A*^ on fibrin clot formation. The parameter values are summarized in table 1.

**Table 1.** Nomenclature for the parameters and variables.

symbol	physical meaning	values (dimension)	reference
*a*	fibrinogen radius	8 nm	[42,43]
$r_{0}^{S}$	reference stretch length	22.5 nm	[11]
*a*_*th*_	threshold of aggregation length	2$r_{0}^{S}$	—
$r_{0}^{A}$	reference aggregation length	2*a*	—
$r_{0}^{R}$	reference repulsive length	2*a*	—
*θ*_0_	reference bending angle	*π*	[10]
*ϕ*_0_	reference torsion angle	0	—
*k*_*b*_	Boltzmann constant	1.38 × 10^−23^ J K^−1^	—
*T*	temperature	300 K	—
*k*^*A*^	aggregation energy constant	0.2–20 × 10^−3^ N m^−1^	[36,37]
*k*^*B*^	bending energy constant	0.1–10 × 10^−18^ J rad^−2^	[25,33]
*k*^*R*^	repulsive energy constant	1.0 × 10^−3^ N m^−1^	—
*k*^*S*^	stretch energy constant	0.01 N m^−1^	[10,32,33]
*k*^*T*^	torsion energy constant	1.0 × 10^−23^ J rad^−2^	[33,34]

### 2.2. Discretization

Following the study by Ermak & McCammon [44], the discrete form of equation (2.2) is rewritten as

2.11 $$c{\dot{r}}_{i}=-\frac{\partial W}{\partial r_{i}}+\frac{\sigma }{\sqrt{\Delta t}}\hat{j},$$

where $\hat{j}$ ( ∈ [0, 1]) is the probability density function following equation (2.10). Based on a uniform random number between 0 and 1 that is given by the XORWOW method [45], we use the Box–Muller method to obtain the value of $\hat{j}$ that satisfies the Gauss distribution [46]. The *i*th nodal point of the protofibril $r_{i}^{n}$ at the *n* time step is updated by Lagrangian tracking, i.e.

2.12 $${\dot{r}}_{i}=\frac{r_{i}^{n+1}-r_{i}^{n}}{\Delta t}.$$

The Euler method is used for time integration except for the stretch term in equation (2.2), which is solved by the explicit fourth-order Runge–Kutta method.

### 2.3. Numerical conditions and analysis

As an initial state, 1215 straight protofibrils (22 nodes/protofibril) are randomly placed in a cubic domain of size 3 μm × 3 μm × 3 μm. Periodic boundary conditions are imposed for all directions. The protofibrils aggregate with each other as time passes, and eventually attain a steady state where they fluctuate only as a result of thermal energy. All simulation cases reached this steady state condition by 50 ms or later (see also figure 3), and hence simulations were performed during a period of 200 ms. In addition, for each run we performed 10 replica simulations from a different initial fibre configuration to ensure that our simulation of the fibre network did not occur in a local energy minimum far from the global minimum. Although it is known that the physiological condition of human fibrinogen is 3–4.5 mg ml [47], some of experimental observations of fibrin clots using scanning electron microscopy (SEM) were conducted in dilute fibrinogen concentrations such as 0.5 mg ml^−1^ [7,13]. For comparisons between calculated steady or extended fibrin clots with those in experimental observations in, e.g. [7,13,41], a fibrinogen concentration was set to 0.5 mg ml^−1^.

**Figure 2.**
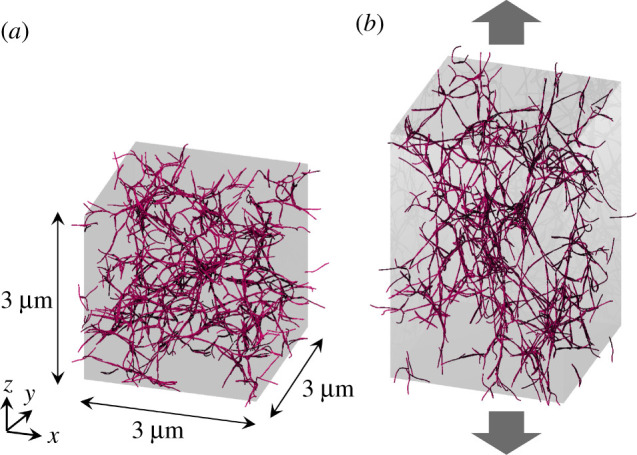
Computational domain consisting of a 3 μm × 3 μm × 3 μm cube. A total of 1215 protofibrils (red rods) are randomly placed and orientated in the initial state. Periodic boundary conditions are imposed for all directions. (*a*) Snapshots of fibrin clot in the steady-state (200 ms after the initial state) and (*b*) fibrin clot extension in the *z*-direction. The results are obtained with *k*^*A*^ = 2 × 10^−3^ N m^−1^ and *k*^*B*^ = 1 × 10^−18^ J rad^−2^.

For steady-state fibrin clots, we also performed stretch testing. Stretching is expressed simply by the proportional scaling of coordinate *z* and the current computational box length *L*_*z*_ for the *z*-direction from current *z* to *ξz* and *L*_*z*_ to *ξL*_*z*_ [39,40] with:

2.13 $$\xi =1+\frac{c_{s}\Delta \tau }{L_{z}},$$

where *c*_*s*_ (=2.5 mm s^−1^) is the stretching speed in the *z*-direction, Δ*τ* (=2 μs) is the relaxation time period. We tested 10 times slower stretching speed (i.e. *c*_*s*_ = 0.25 mm s^−1^), and confirmed that the trajectory of the total energy state against the strain ɛ_*z*_ shown in figure 6*a* did not change. The nodal position for lateral directions (i.e. *x*- and *y*-directions) is not scaled during the extension. The extension is applied until the *z*-directional engineering strain $\varepsilon_{z}=L_{z}/L_{z}^{0}-1$ reaches 1.5, where *L*_*z*_ is the present length of the computational domain and $L_{z}^{0}$ is the initial computational length for the *z*-direction (=3 μm). Since we assume inertia-less protofibril dynamics represented as equation (2.1), and also ensure that fibrin clot dynamics have reached the equilibrium state during Δ*τ*, we can assume that the effect of stretch extension speed on the fibrin clot conformation is negligible. The engineering stress *σ*_*z*_ on the stretch direction (*z*-direction) is calculated from the energy state during fibre extension:

2.14 $$\sigma_{z}=\frac{F_{z}}{A}=\frac{\partial W}{\partial L_{z}}\frac{1}{A},$$

where A (=3 × 3 μm^2^) is the cross-sectional area (*x*–*y* plane) and *F*_*z*_ is the force. A representative snapshot of aggregated protofibrils in the steady state (*t* = 200 ms) and its stretched fibrin clot formation are shown in figure 2*a* and 2*b*, respectively. These results are obtained with *k*^*A*^ = 2 × 10^−3^ N m^−1^ and *k*^*B*^ = 1 × 10^−18^ J rad^−2^.

The orientations of individual protofibrils are defined as

2.15 $$\langle \theta \rangle =\frac{1}{N}\sum_{i}^{N}\langle \theta_{i}\rangle ,$$

2.16 $$\langle \theta_{i}\rangle =\frac{\sum_{i,j}\theta_{i}^{j}l_{i}^{j}}{\sum_{i,j}l_{i}^{j}}$$

2.17 $$\;\mathrm{and}\;\theta_{i}^{j}=\text{arccos}\left(\frac{e_{z}\cdot l_{i}^{j}}{l_{i}^{j}}\right),$$

where $\theta_{i}^{j}(\in [0,\pi /2])$ and $l_{i}^{j}(=|l_{i}^{j}|)$ are the orientation angle and protofibril length of the *j*th segment (*j* ∈ [1, 21]) in the *i*th protofibril (*i* ∈ [1, 1215]), respectively, **e**_*z*_ is a unit vector along the *z*-direction in Euclidean space, 〈 · 〉 denotes the ensemble average and *N* is the total number of protofibrils. Therefore, $\theta_{i}^{j}=0$ indicates that the protofibril is oriented in the *z*-direction. The probability of the orientation of the *i*th protofibril 〈*θ*_*i*_〉 should be normalized by the small band area Δ*S* on the unit sphere, and can be defined as

2.18 $$\begin{array}{rl} & \mathrm{Prob}.{|}_{\left\langle \theta_{i}\rangle \in [\Theta ,\Theta +\Delta \Theta \right]}\\ & \;=\frac{1}{N}\sum_{i}^{N}\frac{\mathrm{Count}.\{\langle \theta_{i}\rangle \;|\;[\Theta ,\Theta +\Delta \Theta ]\}}{\Delta S}\\ & \;=\frac{1}{N}\sum_{i}^{N}\frac{\mathrm{Count}.\{\langle \theta_{i}\rangle \;|\;[\Theta ,\Theta +\Delta \Theta ]\}}{\int_{\Theta }^{\Theta +\Delta \Theta }2\pi \sin\theta \;d\theta }. \end{array}$$

For instance, the probability of aligned protofibrils with orientations of ∈[0, *π*/12] is defined as

2.19 $$\begin{array}{rl} & \mathrm{Prob}.{|}_{\left\langle \theta_{i}\rangle \in [0,\pi /12\right]}\\ & \;=\frac{1}{N}\sum_{i}^{N}\frac{\mathrm{Count}.\{\langle \theta_{i}\rangle |[0,\pi /12]\}}{1-\cos\left(\pi /12\right)}. \end{array}$$

## 3. Results

### 3.1. Aggregation of protofibrils

First, we investigated the spontaneously aggregated form of protofibrils (i.e. no external force) for different *k*^*A*^ and *k*^*B*^. An example of the temporal history of each energy state is shown in figure 3*a*, which was obtained with *k*^*A*^ = 2 × 10^−3^ N m^−1^ and *k*^*B*^ = 1 × 10^−18^ J rad^−2^. The energy state reached a plateau at a relatively early time period of approximately 50 ms. The results regarding fibre conformation, as quantified by the orientation angle, length and tortuosity of individual protofibrils, are shown in figure 3*b*–*d*, respectively. Here, the tortuosity (=*L*/*L*_0_ ≥ 1) is defined as the ratio between the length of protofibril *L* and the Euclidean distance from the start to the end points of protofibril *L*_0_. If the fibre is oriented in the length direction, the tortuosity is *L*/*L*_0_ = 1. These results showed that steady conformation has been reached within 50 ms, and the results are consistent with other cases. Hence, the time average is hereafter uniformly calculated over 100 ms after *t* = 50 ms for all simulations to reduce the influence of the initial conditions.

**Figure 4.**
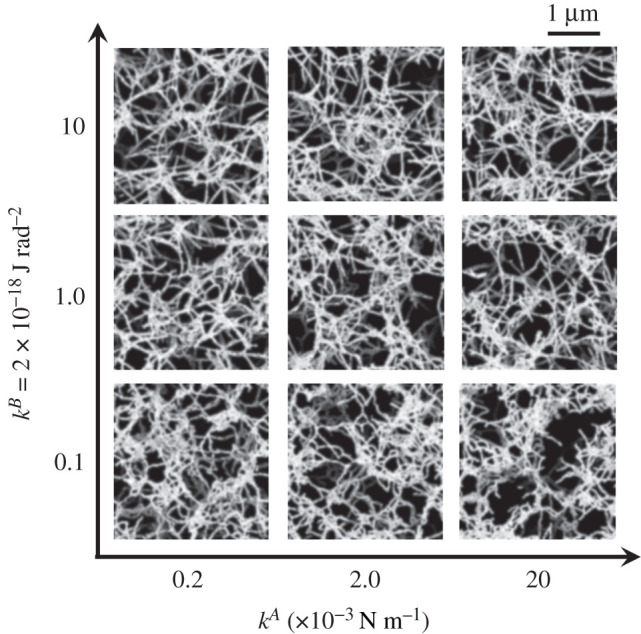
Representative snapshots of fibrin clots at the steady state for different *k*^*A*^ (=0.2, 2 and 20 × 10^−3^ N m^−1^) and *k*^*B*^ (=0.1, 1 and 10 × 10^−18^ J rad^−2^). The scale bar length is 1 μm.

**Figure 5.**
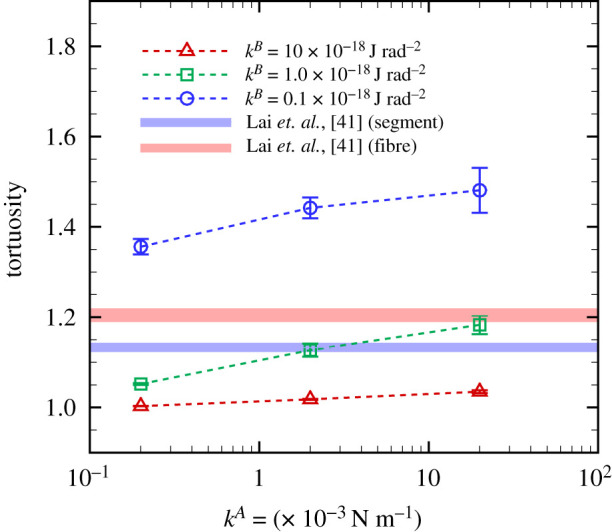
Time average of the tortuosity of individual protofibrils as a function of *k*^*A*^ for different *k*^*B*^ (M ± s.d., run cases N=10). Experimental data [41] of tortuosity of segments and aggregated fibres in collagen-fibrin co-gels with PBS are also displayed.

For comparison with a SEM image [13] and calculated three-dimensional fibrin clots, we projected steady-state fibrin clots onto a two-dimensional plane (*x*–*y* plane at *z* = 0) with 10 nm pixel^−1^. Here, we assigned 10 different luminosity values to the nodes within every 0.3-μm segment in the height *z*-direction, with higher luminosity associated with increased *z*-position. For these projected networks, we applied the Sobel filter to obtain the configuration of protofibrils from calculated nodal points, and also apply the Gaussian filter to obtain smoothed fibre structures. The stack images of steady fibrin clots for different *k*^*A*^ and *k*^*B*^ were obtained using the aforementioned process and are shown in figure 4. Although there was no significant difference in fibre conformation for different *k*^*A*^, the fibres tended to be straighter as *k*^*B*^ increased, a finding that is quantitatively shown in figure 5.

Protofibril conformation was quantified by the tortuosity of individual protofirbrils. The time average of the tortuosity is shown in figure 5, where error bars represent standard deviation of the run cases (M ± s.d., N=10). The results show that at each specific *k*^*A*^, the tortuosity decreases (i.e. fibre linearity is enhanced) as *k*^*B*^ increases, which qualitatively agrees with previous experimental findings that fibre linearity was positively associated with individual protofibrils’ bending resistance [13,17,22]. Experimental data of collagen-fibrin co-gels in phosphate-buffered saline (PBS) are also displayed [41], as quantified from SEM images. Here, in [41], ‘fibres’ are defined as multiple adjacent segments of protofibrils, and a ‘segment’ is the part of a fibre between cross-links. Especially at *k*^*A*^ = 2 × 10^−3^ N m^−1^ and *k*^*B*^ = 1 × 10^−18^ J rad^−2^, calculated tortuosity is highly consistent with the experimental result of a ‘segment’ in [41]. Since SEM requires dehydration, a stricter comparison may be needed to consider the effects of such an imaging process, but this is beyond the scope of the present work.

### 3.2. Stretch tests of fibrin clots

**Figure 6.**
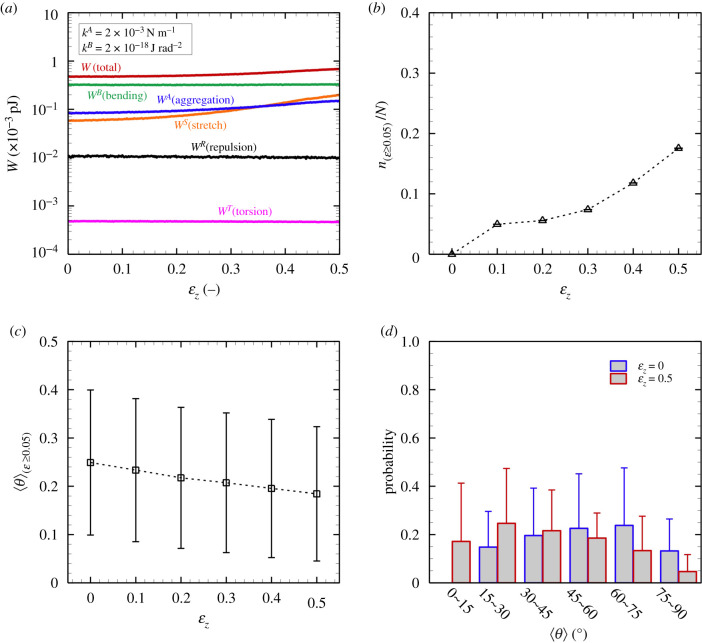
(*a*) Each averaged energy state (without the standard deviation of run cases), (*b*) the ratio of the stretched fibres with *ɛ* ≥ 0.05 *n*_(*ɛ*≥0.05)_/*N*, and (*c*) the ensemble average of the orientation angle of protofibrils with *ɛ* ≥ 0.05 〈*θ*〉_(*ɛ*≥0.05)_, as a function of the engineering strain *ɛ*_*z*_. (*d*) Probability of the orientation angle of protofibrils at *ɛ*_*z*_ = 0 (blue) and *ɛ*_*z*_ = 0.5 (red). The results were obtained with *k*^*B*^ = 1 × 10^−18^ J rad^−2^ and *k*^*A*^ = 2 × 10^−3^ N m^−1^ (M ± SD, N=10).

Next, we performed stretch tests for the aforementioned fibrin clots under the same parameter ranges: *k*^*A*^ = 0.2–20 × 10^−3^ N m^−1^ and *k*^*B*^ = 0.1–10 × 10^−18^ J rad^−2^. An example of the energy state for each engineering strain is shown in figure 6*a*. The result shown in figure 6*a* was obtained with *k*^*A*^ = 2 × 10^−3^ N m^−1^ and *k*^*B*^ = 1 × 10^−18^ J rad^−2^. As the *z*-directional engineering strain *ɛ*_*z*_ increased, the stretch energy *W*^*S*^ gradually increased, and then finally overcame the aggregation energy *W*^*A*^ (figure 6*a*). This tendency was more obvious when *k*^*A*^ increased (data not shown). The decrease of the contribution of *W*^*A*^ to the total energy could be explained by the simple approximation of *W*^*A*^/(*W*^*A*^ + *W*^*S*^) = 1/(1 + *k*^*A*^/*k*^*S*^) if the aggregated protofibrils were oriented to the *z*-direction.

**Figure 7.**
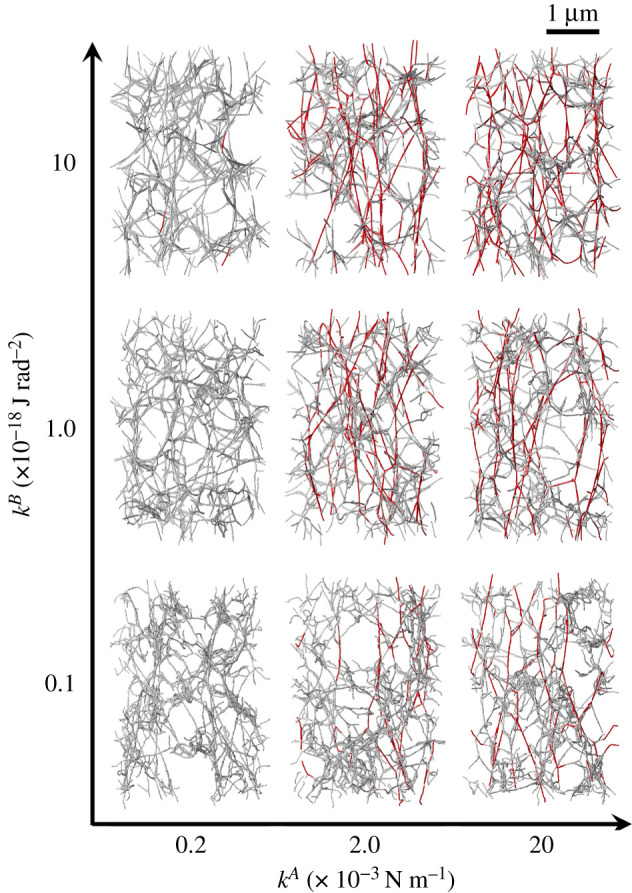
Snapshots of extended fibrin clots at *ɛ*_*z*_ = 0.5 for different *k*^*A*^ and *k*^*B*^. The scale bar length is 1 μm. Protofibrils coloured red are extended beyond the fibre strain *ɛ* (=*l*/*l*_0_ − 1) ≥ 0.05: *l* and *l*_0_ are the current length and at the steady-state length, respectively.

Stretched fibres for different *k*^*A*^ and *k*^*B*^ are shown in figure 7, where protofibrils coloured red are extended beyond the fibre strain *ɛ* (=*l*/*l*_0_ − 1) ≥ 0.05 (*l* and *l*_0_ are the current length and the steady-state length of protofibril, respectively). The number of these extended fibres clearly increased when *k*^*A*^ increased from 0.2 × 10^−3^ to 2 × 10^−3^ N m^−1^ (figure 7). The structures of stretched protofibrils are quantified by the number of such protofibrils, the fibre orientation *θ*, and the probability (Prob.) of the orientation. The ratio of the stretched fibres with *ɛ* ≥ 0.05 to the total number of protofibrils *n*_(*ɛ*≥0.05)_/*N* is shown in figure 6*b*. The percentage of fibres with *ɛ* ≥ 0.05 was only 5% at *ɛ*_*z*_ = 0.2, but it increased to 20% at *ɛ*_*z*_ = 0.5. These fibres’ orientations gradually decreased, i.e. the fibres became oriented to the stretch direction, as shown in figure 6*c*. Since the range of SD almost remains for *ɛ*_*z*_, the result indicates that a distribution of fibre orientation angles exists even under extension. The probability of each orientation was therefore examined, and the results at *ɛ*_*z*_ = 0 and 0.5 are shown in figure 6*d*. At the steady state (*ɛ*_*z*_ = 0), the percentage of fibres oriented towards $\langle \theta \rangle \leq 45^{\circ }$ was less than 50%, although it surpassed 60% at *ɛ*_*z*_ = 0.5 (figure 6*d*).

**Figure 8.**
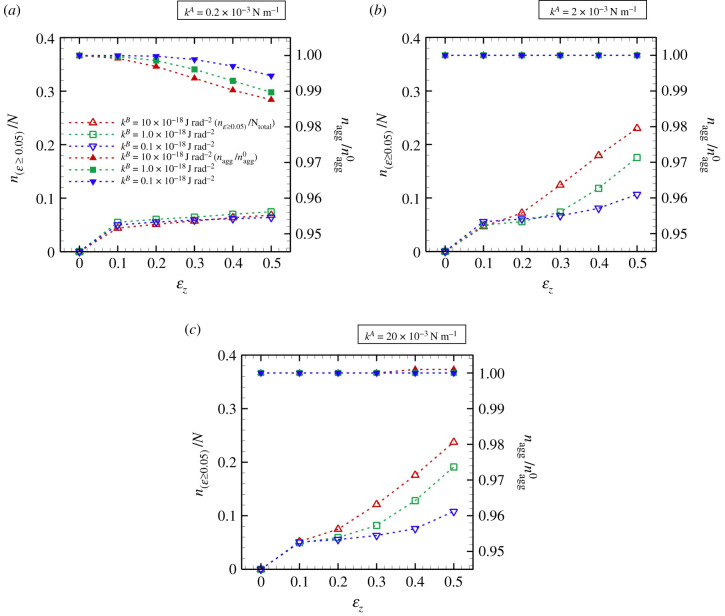
The ratio *n*_(*ɛ*≥0.05)_/*N*, and the ratio of the number of aggregated points *n*_agg_ to the number of initial aggregated points $n_{\mathrm{agg}}^{0}$ ($n_{\mathrm{agg}}/n_{\mathrm{agg}}^{0}$) as a function of *ɛ*_*z*_ for (*a*) *k*^*A*^ = 0.2 × 10^−3^ N m^−1^, (*b*) *k*^*A*^ = 2 × 10^−3^ N m^−1^ and (*c*) *k*^*A*^ = 20 × 10^−3^ N m^−1^, respectively (M ± s.d., N=10).

The population of effectively stretched and aggregated fibres was investigated, and the results are shown in figure 8. At the lowest *k*^*A*^ (=0.2 × 10^−3^ N m^−1^), the ratio *n*_(*ɛ*≥0.05)_/*N* remained almost constant independent of *k*^*B*^ during extension (figure 8*a*). The aggregated fibres started to dissociate for *ɛ*_*z*_ ≥ 0.1 (figure 8*a*), and the dissociation rate was approximately 1%, i.e. $n_{\mathrm{agg}}/n_{\mathrm{agg}}^{0}∼0.99$ at *ɛ*_*z*_ ≥ 0.5, where *n*_agg_ is the number of aggregated points, and $n_{\mathrm{agg}}^{0}$ is the number of initial aggregated points. For relatively large *k*^*A*^ ( ≥2 × 10^−3^ N m^−1^), the ratio *n*_(*ɛ*≥0.05)_/*N* increased with *k*^*B*^, and the fibres remained aggregated even at *ɛ*_*z*_ = 0.5, i.e. $n_{\mathrm{agg}}/n_{\mathrm{agg}}^{0}=1$ (figure 8*b*,*c*). Furthermore, there was no marked difference in *n*_(*ɛ*≥0.05)_/*N* until *ɛ*_*z*_ ≤ 0.2, independent of *k*^*A*^ (figure 8*a*–*c*). Note that, at the largest *k*^*A*^ (=20 × 10^−3^ N m^−1^), only less than 1% fibres still aggregated even for *ɛ*_*z*_ = 0.5 (figure 8*c*).

**Figure 9.**
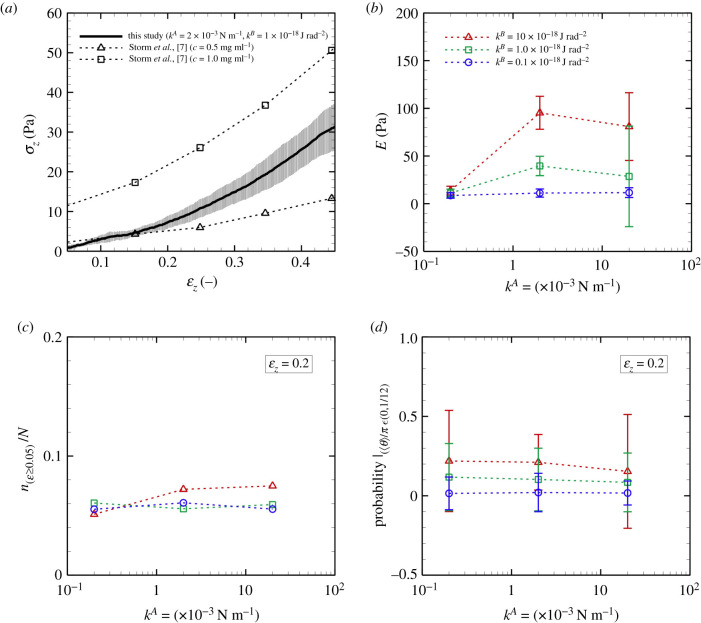
(*a*) Stress *σ*_*z*_ of the fibrin clot as a function of the strain *ɛ*_*z*_. The result is obtained with *k*^*A*^ = 2 × 10^−3^ N m^−1^ and *k*^*B*^ = 1 × 10^−18^ J rad^−2^. Experimental data of the shear modulus of fibrin clots (fibrinogen concentration of 0.5 and 1.0 mg ml^−1^) [7] are also displayed. (*b*) Elastic coefficient *E* of fibrin clots as a function of *k*^*A*^ for different *k*^*B*^. Using the data of *ɛ*_*z*_ = 0.05 to 0.15, *E* was calculated by least-squares fitting to the plot of {*σ*_*z*_ − (*Eɛ*_*z*_ + *σ*_0_)}, where *σ*_0_ is the residual stress. (*c*) Ratio *n*_(*ɛ*≥0.05)_/*N*. (*d*) Probability with 〈*θ*〉 ∈ [0, *π*/12] at *ɛ*_*z*_ = 0.2 as a function of *k*^*A*^ for different *k*^*B*^ (M ± s.d., N=10).

Multiple examples of *σ*_*z*_ as a function of *ɛ*_*z*_, obtained with *k*^*A*^ = 2 × 10^−3^ N m^−1^ and *k*^*B*^ = 1 × 10^−18^ J rad^−2^, are shown in figure 9*a*. The moving-averaged data were obtained for the data of total energy *W* with a window size of *ɛ*_*z*_ = 0.08. We also evaluated different window sizes with a moving-average (*ɛ*_*z*_ = 0.04 and 0.16), and confirmed that there was almost no difference in results between obtained with *ɛ*_*z*_ = 0.04 and *ɛ*_*z*_ = 0.08. The values of *σ*_*z*_ shown in figure 9*a* indicate strain hardening, which qualitatively agrees with experimental data derived with fibrinogen concentrations of 0.5 and 1.0 mg ml^−1^ [7] when an isotropic network model was used. Note that for the lowest *k*^*A*^, the clots showed strain softening for *ɛ*_*z*_ ≥ 0.2 due to dissociation, as shown in figure 8*a*.

Especially for *ɛ*_*z*_ < 0.2, we confirmed an almost proportional relationship between *σ*_*z*_ and *ɛ*_*z*_ in all conditions that were investigated (*k*^*A*^ = 0.2–2 × 10^−3^ N m^−1^ and *k*^*B*^ = 1 × 10^−18^ J rad^−2^), i.e. the effect of dissociation of aggregated fibres on *σ*_*z*_ was negligible. Thus, using the data of *ɛ*_*z*_ = 0.05 to 0.15, the elastic coefficient of fibrin clots *E* was calculated by least-squares fitting [48] to the plot of {*σ*_*z*_ − (*Eɛ*_*z*_ + *σ*_0_)}, where *σ*_0_ is the residual stress. The calculated *E* for different *k*^*A*^ and *k*^*B*^ is summarized in figure 9*b*. Although there was no marked difference in *E* at the smallest *k*^*A*^ (=0.2 × 10^−3^ N m^−1^), *E* increased as *k*^*A*^ rose from 0.2 × 10^−3^ to 2 × 10^−3^ N m^−1^, and the rate of its increase decreased thereafter. the error bars were relatively large, especially at the largest *k*^*A*^ (=20 × 10^−3^ N m^−1^), representing variations of the energy state depending on the initial (quasi-steady) state. For each specific *k*^*A*^, the value of *E* became large with *k*^*B*^, which qualitatively agrees with the following conclusion based on experimental studies [13,17,22]: the bending resistance of individual protofibrils enhances not only fibre linearity but also the elasticity of fibrin clots.

Figure 9*c* shows the ratio *n*_(*ɛ*≥0.05)_/*N* at *ɛ*_*z*_ = 0.2 as a function of *k*^*A*^, and indicates that the number of extended fibres with *ɛ*_*z*_ ≥ 0.05 was insensitive to *k*^*A*^. Figure 9*d* shows the probability of fibres oriented in the stretch direction, defined as those with 〈*θ*〉 ∈ [0, *π*/12] (i.e. Prob.|〈*θ*〉/*π* ∈ [0, 1/12]) at *ɛ*_*z*_ = 0.2. These fibres continued to be oriented in this direction even though *k*^*A*^ increased, while the probability of this orientation increased with *k*^*B*^, i.e. the fibrin clot structure was transformed to one that withstood extension as a result of increased *k*^*B*^ (figure 9*d*). Hence, the attenuation of the rate of increase in *E* with increasing *k*^*A*^ (figure 9*b*) may be correlated with the slow increase of effectively stretched fibres, which are defined with *ɛ* ≥ 0.05 and 〈*θ*〉 ∈ [0, *π*/12].

## 4. Discussion and conclusion

Despite a number of studies of fibrin clots, much is still unknown about the relationship between the behaviour of individual protofibrils and the macro-scale mechanical response of fibrin clots. Yesudasan *et al.* proposed the coarse-grained molecular dynamics model, featuring several hundred nanometre-scale structures, to investigate a complex network of fibrin clots in terms of fibrinogen molecules [25,26]. However, there remain limitations to the mesoscopic mode focusing on the dynamics of fibrin clots, whose interactions can be characterized on a cellular scale (i.e. several micrometres). Stylianopoulos & Barocas developed a structural model that uses volume-averaging theory to study the mechanical behaviour of collagen networks [49], wherein the bending rigidity of individual fibres was neglected. Although their model successfully demonstrated the strain-hardening character of type I collagen extracellular matrices observed experimentally [50], other experimental studies using X-rays [51] or mechanical (shear strain) testing [52] suggested that the flexibility of fibrin clots may arise from bending of the fibres, a theory that was supported by later studies using electron microscopy [53] and optical tweezers [17]. Despite the insights about bending properties at the fibre level, the relationship between such mechanical properties on individual fibre conformations and the mechanical responses of clots are still unknown. To tackle these challenges, a mesoscopic framework based on protofibril behaviours is necessary, but has not yet been established. We thus propose a minimal mesoscopic model for protofibrils based on Brownian dynamics. Through various numerical simulations, we evaluated the feasibility of our mesoscopic model analysis.

The approach used in our model successfully demonstrated conformation of a fibrin clot (figure 5) which was consistent with experimental data in [13,41]. Further, our numerical results captured the strain-hardening response, e.g. as reported in [7] based on protofibril dynamics (figure 9*a*). Although there are a number of experimental works about tensile mechanical properties in living materials [54] and also single-molecule biophysics [55], there are few reports about assessments of the stress acting on fibrin clots during extensions. For instance, Roeder *et al.* performed tensile mechanical tests for matrices purified from type I collagen for different collagen concentrations ranging between 0.3–3 mg ml^−1^, and showed the strain-hardening character of the matrices [50]. The experimental estimation of the engineering stress obtained with 2 mg ml^−1^ collagen concentration was one or two orders of magnitude higher (i.e. *O*(*σ*_*z*_) = 10^2^–10^3^ Pa) in the range between 10–20%-strain than our numerical results of fibrin clots with 0.5 mg ml^−1^ fibrinogen concentration [50].

As with other numerical models, our mesoscopic model required a number of parameters. Although there are many SEM results regarding human fibrin clot structures (e.g. [16]), quantitative data of fibrin fibre parameters are still limited, and thus we could not determine them in a straightforward manner. Yesudasan *et al.* [25,26] used mathematical methods [56] to characterize and quantify 3D images of fibrin clot networks, and then compared them with simulation results [25]. Although our model parameters were not determined by this type of bottom-up molecular dynamic approach, our methodology potentially characterized the conformation of fibrin clots, as well as their mechanical responses, according to the model parameters. We thus suggest that our microscopic model can be used to estimate the relationship between the nanometre-scale dynamics of individual protofibrils and the mechanical properties of micrometre-scale fibrin clots. For example, simulations of aggregated protofibrils whose degree of extension depends on the aggregation energy constant *k*^*A*^ will provide insight into the dependency of factor XIIIa on fibrin clot elasticity [13,18,19]. Since our numerical results showed that the elasticity of the fibrin clots increased with *k*^*A*^, the dependence of factor XIIIa on the elasticity resulting from the fibrous architecture of the clots may be characterized by *k*^*A*^. Previous experimental results showed that stiffening of fibrin clots can result from the response of the polymeric protofibrils between cross-links, from alterations in the network structure, or both [6,7,14,15]. Although we did not fully quantify the architecture of aggregated protofibrils, the simulated protofibrils in the networks also tended to align in the stretch direction as *k*^*B*^ increased (figure 9*d*), resulting in sparse networks (figure 7). Such interlinked protofibril structures may contribute to decreasing the elasticity of fibrin clots, especially at the highest *k*^*A*^ (=2.0 × 10^−3^ N m^−1^) and *k*^*B*^ (=1.0 × 10^−18^ J rad^−2^) (figure 9*b*). Future quantitative analysis of the network structure will provide insight into the relationship between the fibrous architecture and mechanical properties of the clot. Experimental observations using SEM have clarified the precise structure of fibrin clots, while some limitations should be carefully considered. SEM analysis allows us to visualize single fibres even under load [57], but originally hydrated fibrin networks are most often dehydrated and fixed, and hence it remains difficult to capture 3D networks of fibrin clots in dynamics. Spectroscopic tools such as Fourier transform infrared (FTIR) and Raman scattering offer alternative methods to probe structural changes of proteins using molecular vibrations. These methods can be performed on 3D fibrin hydrogels under mechanical deformation *in situ*. For instance, Litvinov *et al.* used FTIR spectroscopy to reveal force-induced changes in the secondary structure of hydrated fibrin clots made of human blood plasma *in vitro* [58]. In more recently, Wang *et al.* used broadband coherent anti-Stokes Raman scattering (BCARS) microscopy and custom-built loading devices to capture the molecular vibrational signature of fibrin under shear and tensile deformations *in situ* [59]. Along with those microscopic observation techniques, image analyses of a 3D network of fibrin clots obtained with confocal microscopy have been reported as well as its observation techniques [60,61]. The analyses have also gained insights into the mechanics of a fibrin network in terms of its microscopic structure [60,61].

Venous thromboembolism, including deep-vein thrombosis and pulmonary embolism, is the leading cause of lost disability-adjusted life years and the third leading cause of cardiovascular death in the world [62]. Potential risk analysis for thromboembolism is of fundamental importance in diagnosis or therapeutic decisions. Recent experimental observations showed that there are differences in structure and composition between arterial and venous thrombi and pulmonary emboli [63]. Therefore, the tensile mechanical properties of those thrombi characterized as volume fraction of fibrin fibres or fibrin bundles would be one of risks in thromboembolism. Since our numerical model enables the investigation of tensile stress as well as elastic coefficient for different fibrinogen concentrations, calculated elastic coefficients of fibrin clots with specific fibrinogen concentrations based on blood samples would provide additional bioengineering insights in the diagnosis of thromboembolism. While nonlinear mechanical properties of fibrin clots can be useful in bioengineering applications not only in wound repair but also drug delivery, cell delivery, cell differentiation, tissue engineering and patterning [59,64,65]. Our numerical model will gain insights into their designs, in terms of mechanical properties.

In *in vitro* experiments, clots are often obtained under conditions of purified fibrinogen or plasma with low platelet counts. However, in the actual human circulation, haemostatic clots or obstructive thrombi are formed in the presence of blood flow that profoundly affects the formation of the fibrin network and its structure and properties. For instance, it is known that the mechano-sensitive plasma protein von Willebrand factor (vWF) and its binding with the platelet receptor glycoprotein Ib*α* (GPIb*α*) play a crucial role in haemostasis, yet is also key to pathological thrombus initiation and propagation, which are well reviewed in, e.g., [62,66]. Furthermore, the circulation continually brings more fibrinogen to the developing clot, and as a result it becomes denser, with thicker, bundled fibres [67]. Fluid flow can also cause the fibrin fibres to orient along the direction of flow both *in vitro* [68,69] and *in vivo* [70], which has important consequences for the mechanical properties of the clots [69,71]. Flow fields would also affect not only the structure of thrombi but also the composition. Recent clinical observation using high-resolution SEM clearly showed that venous thrombi are fibrin-rich, and arterial thrombi are fibrin- and platelet-rich [63]. The evidence that arterial thrombi contain a large amount of fibrin [63] is in contrast to previous animal experiments by [72,73], where high wall shear rates over the range 50–2500 s^−1^ impede fibrin deposition in subendothelium [72] and extracellular matrix [73]. Hence, further investigations about mechanical factors regulating thrombus growth are needed while paying attention to haemodynamic flow as well as mechanical reactions of plasma proteins. In our simulation, we did not consider the flow of background medium, and also neglected the presence of red cells/platelets and solvent proteins. Since there are only a few reports on the modelling of protein activation under cellular blood flow [74–76], it would be interesting to study how fibrous interactions affect the suspension rheology of red blood cells (RBCs) [77] or the flow behaviours of RBCs and platelets in microvessels depending on shear rates [38,78].

In summary, we proposed a minimal mesoscopic model of protofibrils based on Brownian dynamics. Since our results successfully captured the conformation of aggregated protofibrils (e.g. tortuosity [41]) as well as their mechanical response (e.g. strain-hardening response [7]) depending on model parameters, we suggest that our microscopic model approach can estimate the relationship between nanometre-scale dynamics of individual protofibrils and the mechanical properties of micrometre-scale fibrin clots. For example, the dependence of specific mediators such as factor XIIIa on the network architecture, and thus on the elasticity of fibrin clots, can be estimated by modelling the aggregation or bending stiffness on the level of protofibril fibres. Our numerical results suggest that the aggregated protofibril structure was altered to withstand extension by increasing the bending stiffness of individual protofibrils, which is consistent with experimental results [13,17,22]. In the future, we will report the relationship between individual protofibril behaviours and the macro-scale mechanical response of fibrin clots, including the fibrous network structure.

## Data accessibility

The data used in the figures are provided in the electronic supplementary material.

## Appendix A

**A.1. Bending energy**

For equations (2.4*c*), we redefine the position vectors as

A 1*a* $$p\equiv r_{j},$$

A 1*b* $$q\equiv r_{i}$$

A 1*c* $$\;\mathrm{and}\;r\equiv r_{k},$$

and also

A 2*a* $$a\equiv r_{ij}=r_{j}-r_{i}=p-q$$

and

A 2*b* $$b\equiv r_{ik}=r_{k}-r_{i}=r-q.$$

Then, the bending energy can be expressed as

A 3*a* $$W^{B}=\sum_{e\in D^{B}}\Delta W_{e}^{B},$$

A 3*b* $$\Delta W_{e}^{B}=\frac{k^{B}}{2}{\left(\phi_{e}-\phi_{0}\right)}^{2}$$

A 3*c* $$\;\mathrm{and}\;\phi_{e}=\text{arccos}\left(\frac{a\cdot b}{\left|a∥b\right|}\right),$$

where $D^{B}$ is the total set of indices for bending elements. The nodal force $f_{i}^{B}$ (*i* ∈ [1, *N*]; *N* is the number of nodal points) is calculated by

A 4*a* $$f_{i}^{B}=-\frac{\partial W^{B}}{\partial r_{i}}=\sum_{e\in \partial D_{i}^{B}}\Delta f_{e}^{B}$$

and

A 4*b* $$\Delta f_{e}^{B}=-\frac{\partial \Delta W_{e}^{B}}{\partial \phi_{e}}\frac{\partial \phi_{e}}{\partial r_{i}},$$

where $\partial D_{i}^{B}$ is the subset of $D^{B}$ involving the nodal point **r**_*i*_. Focusing on the formulation of a bending element *e*, we calculate as

A 5 $$\frac{\partial \Delta W^{B}}{\partial \phi }=k^{B}(\phi_{e}-\phi_{0}).$$

and

A 6*a* $$\frac{\partial \phi }{\partial p}=\frac{\partial a}{\partial p}\cdot \frac{\partial \phi }{\partial a}+\frac{\partial b}{\partial p}\cdot \frac{\partial \phi }{\partial b}=\frac{\partial \phi }{\partial a},$$

A 6*b* $$\frac{\partial \phi }{\partial q}=\frac{\partial a}{\partial q}\cdot \frac{\partial \phi }{\partial a}+\frac{\partial b}{\partial q}\cdot \frac{\partial \phi }{\partial b}=-\frac{\partial \phi }{\partial a}-\frac{\partial \phi }{\partial b}$$

A 6*c* $$\;\mathrm{and}\;\frac{\partial \phi }{\partial r}=\frac{\partial a}{\partial r}\cdot \frac{\partial \phi }{\partial a}+\frac{\partial b}{\partial r}\cdot \frac{\partial \phi }{\partial b}=\frac{\partial \phi }{\partial b}.$$

Introducing Θ=a⋅b/|a∥b|=cos⁡θ, we obtain

A 7 $$\begin{array}{rl} \frac{\partial \phi }{\partial a} & =\frac{\partial \text{arccos}\Theta }{\partial \Theta }\frac{\partial \Theta }{\partial a}\\ & =\frac{-1}{\sqrt{1-\Theta^{2}}}\frac{\partial }{\partial a}\left(\frac{a}{\left|a\right|}\right)\cdot \frac{b}{\left|b\right|}\\ & =-\frac{1}{\sqrt{1-\Theta^{2}}}\left(\frac{1}{\left|a\right|}I-\frac{a}{|a{|}^{3}}a\right)\cdot \frac{b}{\left|b\right|}\\ & =-\frac{1}{\sqrt{1-\Theta^{2}}}\frac{1}{\left|a\right|}\left(\frac{b}{\left|b\right|}-\frac{a}{\left|a\right|}\frac{a\cdot b}{\left|a∥b\right|}\right)\\ & =-\frac{1}{\sin\theta }\frac{1}{\left|a\right|}\left(\frac{b}{\left|b\right|}-\frac{a}{\left|a\right|}\cos\theta \right), \end{array}$$

and analogously

A 8 $$\frac{\partial \phi }{\partial b}=-\frac{1}{\sin\theta }\frac{1}{\left|b\right|}\left(\frac{a}{\left|a\right|}-\frac{b}{\left|b\right|}\cos\theta \right).$$

Consequently, the nodal forces in a bending element *e* are given by

A 9 $$\Delta f_{p}^{B}=-k^{B}(\phi_{e}-\phi_{0})\frac{\partial \phi_{e}}{\partial a},$$

A 10 $$\Delta f_{q}^{B}=k^{B}(\phi_{e}-\phi_{0})\left(\frac{\partial \phi_{e}}{\partial a}+\frac{\partial \phi }{\partial b}\right)$$

A 11 $$\;\mathrm{and}\;\Delta f_{r}^{B}=-k^{B}(\phi_{e}-\phi_{0})\frac{\partial \phi_{e}}{\partial b}.$$

**A.2. Torsion energy**

For equation (2.5*c*), we redefine the position vectors as

A 12*a* $$p\equiv r_{j},$$

A 12*b* $$q\equiv r_{i},$$

A 12*c* $$r\equiv r_{k}$$

A 12*d* $$\;\mathrm{and}\;s\equiv r_{l}.$$

and

A 13*a* $$a\equiv r_{ij}=r_{j}-r_{i}=p-q,$$

A 13*b* $$b\equiv r_{ik}=r_{k}-r_{i}=r-q,$$

A 13*c* $$c\equiv r_{ki}=r_{i}-r_{k}=q-r=-b$$

A 13*d* $$\;\mathrm{and}\;d\equiv r_{kl}=r_{l}-r_{k}=s-r.$$

Then, we rewrite equation (2.6) as

A 14*a* m=a×b

and

A 14*b* n=c×d.

The torsion energy *W*^*T*^ is given by

A 15*a* $$W^{T}=\sum_{e\in D^{T}}\Delta W_{e}^{T},$$

A 15*b* $$\Delta W_{e}^{T}=\frac{k^{T}}{2}{\left(\phi_{e}-\phi_{0}\right)}^{2}$$

A 15*c* $$\;\mathrm{and}\;\phi_{e}=\text{arccos}\left(\frac{m\cdot n}{\left|m∥n\right|}\right),$$

where $D^{T}$ is the total set of indices for torsional elements. The nodal force $f_{i}^{T}$ (*i* ∈ [1, *N*]) is calculated by

A 16*a* $$f_{i}^{T}=-\frac{\partial W^{T}}{\partial r_{i}}=\sum_{e\in \partial D_{i}^{T}}\Delta f_{e}^{T}$$

and

A 16*b* $$\Delta f_{e}^{T}=-\frac{\partial \Delta W_{e}^{T}}{\partial \phi_{e}}\frac{\partial \phi_{e}}{\partial r_{i}},$$

where $\partial D_{i}^{T}$ is the subset of $D^{T}$ involving the nodal point **r**_*i*_. Focusing on the formulation of a torsional element *e*, we calculate as

A 17 $$\frac{\partial \Delta W_{e}^{T}}{\partial \phi }=k^{T}(\phi_{e}-\phi_{0})$$

and

A 18 $$\frac{\partial \phi }{\partial \alpha }=\frac{\partial m}{\partial \alpha }\cdot \frac{\partial \phi }{\partial m}+\frac{\partial n}{\partial \alpha }\cdot \frac{\partial \phi }{\partial n},\;(\alpha =p,q,r,s).$$

Introducing Θ=m⋅n/|m∥n|=cos⁡θ, and referring to equations (A7) and (A8), we obtain

A 19*a* $$\frac{\partial \phi }{\partial m}=-\frac{1}{\sin\theta }\frac{1}{\left|m\right|}\left(\frac{n}{\left|n\right|}-\frac{m}{\left|m\right|}\cos\theta \right)$$

and

A 19*b* $$\frac{\partial \phi }{\partial n}=-\frac{1}{\sin\theta }\frac{1}{\left|n\right|}\left(\frac{m}{\left|m\right|}-\frac{n}{\left|n\right|}\cos\theta \right).$$

We derive the index notation with the summation convention

A 20*a* $$\begin{array}{rl} \frac{\partial m}{\partial \alpha } & =\frac{\partial ((p-q)\times (r-q))}{\partial \alpha }\\ \Rightarrow \;\frac{\partial m_{j}}{\partial \alpha_{i}} & =\varepsilon_{\;jkl}\frac{\partial ((p_{k}-q_{k})(r_{l}-q_{l}))}{\partial \alpha_{i}} \end{array}$$

and

A 20*b* $$\begin{array}{rl} \frac{\partial n}{\partial \alpha } & =\frac{\partial ((q-r)\times (s-r))}{\partial \alpha }\\ \Rightarrow \;\frac{\partial n_{j}}{\partial \alpha_{i}} & =\varepsilon_{\;jkl}\frac{\partial ((q_{k}-r_{k})(s_{l}-r_{l}))}{\partial \alpha_{i}},\\ & \;(\alpha =p,q,r,s), \end{array}$$

where

A 21*a* $$\begin{array}{rl} \frac{\partial m_{j}}{\partial p_{i}} & =\varepsilon_{\;jkl}\frac{\partial ((p_{k}-q_{k})(r_{l}-q_{l}))}{\partial p_{i}}\\ & =\varepsilon_{\;jkl}\delta_{ik}(r_{l}-q_{l})=\varepsilon_{\;jik}(r_{k}-q_{k}),\\ \frac{\partial m_{j}}{\partial p_{i}} & =\varepsilon_{\;jkl}\frac{\partial ((q_{k}-r_{k})(s_{l}-r_{l}))}{\partial p_{i}}\\ & =0, \end{array}\}$$

A 21*b* $$\begin{array}{rl} \frac{\partial m_{j}}{\partial q_{i}} & =\varepsilon_{\;jkl}\frac{\partial ((p_{k}-q_{k})(r_{l}-q_{l}))}{\partial q_{i}}\\ & =-\varepsilon_{ijk}(p_{k}-q_{k})-\varepsilon_{\;jik}(r_{k}-q_{k}),\\ \frac{\partial n_{j}}{\partial q_{i}} & =\varepsilon_{\;jkl}\frac{\partial ((q_{k}-r_{k})(s_{l}-r_{l}))}{\partial q_{i}}\\ & =\varepsilon_{\;jik}(s_{k}-r_{k}), \end{array}\}$$

A 21*c* $$\begin{array}{rl} \frac{\partial m_{j}}{\partial r_{i}} & =\varepsilon_{\;jkl}\frac{\partial ((p_{k}-q_{k})(r_{l}-q_{l}))}{\partial r_{i}}\\ & =\varepsilon_{ijk}(p_{k}-q_{k}),\\ \frac{\partial m_{j}}{\partial r_{i}} & =\varepsilon_{\;jkl}\frac{\partial ((q_{k}-r_{k})(s_{l}-r_{l}))}{\partial r_{i}}\\ & =-\varepsilon_{ijk}(q_{k}-r_{k})-\varepsilon_{\;jik}(s_{k}-r_{k}), \end{array}\}$$

and

A 21*d* $$\begin{array}{rl} \frac{\partial m_{j}}{\partial s_{i}} & =\varepsilon_{\;jkl}\frac{\partial ((p_{k}-q_{k})(r_{l}-q_{l}))}{\partial s_{i}}\\ & =0,\\ \frac{\partial m_{j}}{\partial s_{i}} & =\varepsilon_{\;jkl}\frac{\partial ((q_{k}-r_{k})(s_{l}-r_{l}))}{\partial s_{i}}\\ & =\varepsilon_{ijk}(q_{k}-r_{k}), \end{array}\}$$

Thus,

A 22*a* $$\begin{array}{rl} & \frac{\partial m}{\partial \alpha }\cdot \beta =\frac{\partial m_{j}}{\partial \alpha_{i}}\beta_{j}e_{j}\\ & \;=\left\{ \begin{array}{cc} b\times \beta , & \left(\alpha =p\right)\\ -\beta \times a-b\times \beta =\beta \times (b-a), & \left(\alpha =q\right)\\ \beta \times a, & \left(\alpha =r\right)\\ 0, & \left(\alpha =s\right) \end{array}\right. \end{array}$$

and

A 22*b* $$\begin{array}{rl} & \frac{\partial n}{\partial \alpha }\cdot \beta =\frac{\partial n_{j}}{\partial \alpha_{i}}\beta_{j}e_{j}\\ & \;=\left\{ \begin{array}{cc} 0, & \left(\alpha =p\right)\\ d\times \beta , & \left(\alpha =q\right)\\ -\beta \times c-d\times \beta =-\beta \times (c-d), & \left(\alpha =r\right)\\ \beta \times c, & \left(\alpha =s\right) \end{array}\right. \end{array}$$

for the arbitrary vector **β**. Consequently, the nodal forces in a torsional element *e* are given by

A 23*a* $$\begin{array}{rl} \Delta f_{p}^{T} & =-\frac{\Delta W^{T}}{\partial \phi }\left(\frac{\partial m}{\partial p}\cdot \frac{\partial \phi }{\partial m}+\frac{\partial n}{\partial p}\cdot \frac{\partial \phi }{\partial n}\right)\\ & =k^{T}(\phi -\phi_{0})\left(\frac{\partial \phi }{\partial m}\times b\right), \end{array}$$

A 23*b* $$\begin{array}{rl} \Delta f_{q}^{T} & =-\frac{\Delta W^{T}}{\partial \phi }\left(\frac{\partial m}{\partial q}\cdot \frac{\partial \phi }{\partial m}+\frac{\partial n}{\partial q}\cdot \frac{\partial \phi }{\partial n}\right)\\ & =-k^{T}(\phi -\phi_{0})\left(\frac{\partial \phi }{\partial m}\times (b-a)-\frac{\partial \phi }{\partial n}\times d\right), \end{array}$$

A 23*c* $$\begin{array}{rl} \Delta f_{r}^{T} & =-\frac{\Delta W^{T}}{\partial \phi }\left(\frac{\partial m}{\partial r}\cdot \frac{\partial \phi }{\partial m}+\frac{\partial n}{\partial r}\cdot \frac{\partial \phi }{\partial n}\right)\\ & =-k^{T}(\phi -\phi_{0})\left(\frac{\partial \phi }{\partial m}\times a-\frac{\partial \phi }{\partial n}\times (c-d)\right) \end{array}$$

and

A 23*d* $$\begin{array}{rl} \Delta f_{s}^{T} & =-\frac{\Delta W^{T}}{\partial \phi }\left(\frac{\partial m}{\partial s}\cdot \frac{\partial \phi }{\partial m}+\frac{\partial n}{\partial s}\cdot \frac{\partial \phi }{\partial n}\right)\\ & =-k^{T}(\phi -\phi_{0})\left(\frac{\partial \phi }{\partial n}\times c\right). \end{array}$$

Note that in practical application, we take

A 24 $$\phi =\mathrm{sgn}(b\cdot (m\times n))\text{arccos}\left(\frac{m\cdot n}{\left|m∥n\right|}\right).$$
